# A classification-based approach to low back pain in primary care – protocol for a benchmarking controlled trial

**DOI:** 10.1186/s12875-020-01135-8

**Published:** 2020-04-06

**Authors:** A. S. Simula, A. Malmivaara, N. Booth, J. Karppinen

**Affiliations:** 1 Medical Research Center Oulu, Oulu University Hospital and University of Oulu, Oulu, Finland; 2 Department of General Medicine, The South Savo Social and Health Care Authority, Mikkeli, Finland; 3 Centre for Health and Social Economics, Finnish Institute for Health and Welfare, Helsinki, Finland; 4 Faculty of Social Sciences (Health Sciences), Tampere University, Tampere, Finland; 5 Finnish Institute of Occupational Health, Oulu, Finland

**Keywords:** Low back pain, Biopsychosocial approach, Classification-based approach, Implementation research, Benchmarking controlled study, Primary care, STarT Back tool

## Abstract

**Background:**

Guidelines recommend a biopsychosocial framework for low back pain (LBP) management and the avoidance of inappropriate imaging. In clinical practice, care strategies are often inconsistent with evidence and guidelines, even though LBP is the most common disabling health condition worldwide. Unhelpful beliefs, attitudes and inappropriate imaging are common. LBP is understood to be a complex biopsychosocial phenomenon with many known multidimensional risk factors (symptom- and lifestyle-related, psychological and social) for persistent or prolonged disability, which should be identified and addressed by treatment. The STarT Back Tool (SBT) was developed for early identification of individual risk factors of LBP to enable targeted care. Stratified care according SBT has been shown to improve the effectiveness of care in a primary care setting. A biopsychosocially-oriented patient education booklet, which includes imaging guidelines and information, is one possible way to increase patients’ understanding of LBP and to reduce inappropriate imaging. Premeditated pathways, education of professionals, written material, and electronic patient registry support in health care organizations could help implement evidence-based care.

**Methods:**

We will use a Benchmarking Controlled Trial (BCT) design in our study. We will prospectively collect data from three health care regions before and after the implementation of a classification-based approach to LBP in primary care. The primary outcome will be change in PROMIS (Patient-Reported Outcomes Measurement Information System) (short form 20a) over 12-month follow-up.

**Discussion:**

The implementation of a classification-based biopsychosocial approach can potentially improve the care of LBP patients, reduce inappropriate imaging without increasing health-care costs, and decrease indirect costs by reducing work disability. Using the BCT we will be able to evaluate the effectiveness of the improvement strategy for the entire care pathway.

**Trial registration:**

**ISRCTN,**ISRCTN13273552, retrospectively registered 13/05/2019.

## Background

Guidelines recommend biopsychosocial frameworks for low back pain (LBP) management and avoidance of inappropriate imaging [1–3]. In clinical practice, care strategies are not always aligned with evidence and guidelines even though LBP is the most common disabling health condition worldwide [4, 5]. Unhelpful beliefs, attitudes and inappropriate imaging are common [6, 7]. A specific cause of pain can only be found for a small percentage of LBP patients, and over 90% are classified as having non-specific pain [8]. LBP is a complex condition in which biological, psychological, and social factors impact on both the experience of back pain and the associated disability [3, 9]. Risk factors for poor prognosis of LBP include high pain intensity, adverse subjective belief of long-lasting pain, low self-efficacy (i.e. confidence in one’s own ability to get on with life despite the pain), passive coping strategies, high catastrophizing and fear avoidance beliefs, depression, sleep problems, psychological distress, low education and social class, and unemployment [3, 4, 10–12].

In addition to patient-related factors and management, professional and health care system-related factors also affect the outcomes of patients with LBP. Such factors include imaging policy, patient education, the attitudes and beliefs of health care professionals, and the timing of rehabilitation [2, 13, 14].

Imaging findings of degeneration of the lumbar spine are also prevalent among asymptomatic adults [15, 16]. Imaging of LBP patients without indications of serious underlying conditions does not improve clinical outcomes [17]. On the contrary, early magnetic resonance imaging (MRI) may be even harmful for LBP patients [18, 19]. Early MRI is associated with additional examinations, injections, operations, and increased health care utilization and costs [20, 21]. Adding epidemiologic data to MRI reports may decrease specialist consultations, repeated imaging and narcotics prescriptions, whereas no effect has been shown on the rate of operations and injections [22, 23]. Comprehensive evidence-based information on LBP is more beneficial than imaging. Patient education based on the biopsychosocial model has shown to be an effective strategy for modifying beliefs about LBP, minimizing its consequences and improving treatment compliance [24, 25]. Appropriate patient education can reduce pain and disability in the short term [26]. Even simple patient information has shown to be cost-effective and to produce savings in the costs of managing mild low back symptoms in occupational settings [27].

Practitioners’ beliefs affect how they explain pain to the patient and what kind of care they choose [28, 29]. Non-pharmacological treatment considering psychosocial factors is not given, as LBP guidelines and evidence suggest, if the attitudes and beliefs of the treating physician are biomedical [30]. It also matters which type of health care professional first sees the patient. Direct access to a physiotherapist (PT) reduces waiting times, improves outcomes through earlier access to care, prevents acute problems becoming chronic, reduces long-term pain and disability, and decreases time off work [14, 31]. Direct access to a PT has resulted in high satisfaction among both patients and PTs [14]. In Finland, direct access to a PT due to musculoskeletal symptoms has been observed to reduce the number of general practitioner (GP) visits, with only 4% of patients with direct PT access being referred to a GP [32].

Treatment of LBP should address the complexity of biological, psychological and social factors [33]. The risk of prolonged disability due to pain varies among patients, which can be seen during the first health care visit [34, 35]. Standardized questionnaires can be used for risk identification because professionals’ ability to identify psychosocial risk factors is limited even if they have been trained [36]. The STarT Back Tool (SBT) questionnaire allocates LBP patients to low-, medium- or high-risk groups of persistent disabling back pain [37]. The SBT has been validated in Finnish [38]. Using the SBT as a screening method for the classification-based approach has shown to improve the effectiveness of LBP treatment in primary care [37]. Successful use of a classification-based approach using SBT as a screening method has shown significant improvements in patient disability outcomes, halving time off work without increasing health care costs [39]. On the other hand, if its implementation has been deficient or unfeasible, no effects have been found [40].

In order to be able to provide evidence-based care, it is essential that health care organizations ensure that the correct options are feasible for professionals by, for example, providing them with sufficient knowledge and training [41] and systematically identifying patients’ individual risk factors^35.^ Using classification-based care pathways may enable the optimization of patients’ treatment and the use of limited health care resources. Written local policy information, support of electronic patient registry functions, education of professionals, successful co-operation between professionals, and a patient education booklet supporting evidence-based care could all enhance the implementation and effectiveness of classification-based care for LBP in primary care [42, 43]. Applying a classification-based approach throughout the care pathway is likely to present a major opportunity to enhance cost-effectiveness of LBP treatments in primary care and hence reduce LBP-related disability.

We hypothesize that a classification-based approach, which includes providing the correct information and a new means for professionals to assess and treat LBP patients according to their individual risk profile, will optimize the treatment of patients as well as the use of health care resources. We further assume that the classification-based approach will be effective and save costs in comparison to current best practice.

### Objectives

The aim of this study is to investigate whether the classification-based approach to LBP patients and the education of professionals in primary care improves patients’ functional ability and quality of life and reduces work disability in both the short (over the first 3 months) and long term (over the first year). Furthermore, we aim to evaluate the cost-effectiveness of this approach in comparison to current best practice, using direct costs such as visits to health care professionals, referrals to MRI, and pain medication; and indirect costs such as sick leaves and disability pensions. A secondary objective is to evaluate whether education can change professionals’ attitudes towards LBP and its treatment by following their attitudes, beliefs and satisfaction related to the treatment process.

### Trial design

We will use the Benchmarking Controlled Trial (BCT) as our study design [44]. The BCT enables the evaluation of differences in effectiveness between clinical pathways and complex interventions targeting health care system factors [45]. It also documents and takes into consideration the characteristics of regional health care systems or organizations.

## Methods/ design

### Study setting

We have selected three primary health care regions for the study, which are described in Table 1. First, ESSOTE (Etelä-Savon sosiaali- ja terveyspalvelut, The South Savo social and health care authority), which consists of the city of Mikkeli (population approx. 54,000) and smaller areas around Mikkeli (Hirvensalmi, Mäntyharju, Puumala, Juva, Pertunmaa, Kangasniemi) and has been one joint health care region since 2017. Second, EKSOTE (Etelä-Karjalan sosiaali- ja terveyspiiri, South Karelia social and health care district), which is a joint municipality authority of the South Karelia region, and comprises nine municipalities: Lappeenranta, Lemi, Luumäki, Imatra, Parikkala, Rautjärvi, Ruokolahti, Savitaipale, and Taipalsaari. Of these, the city of Lappeenranta is the largest, with approximately 72,000 inhabitants. Rovaniemi city is the study’s third health care region and contains four health care centres: Pulkamontie, Urheilukatu, Metsäruusuntie and Rinteenkulma (population covered approx. 62,000). All general physicians and PTs in the study’s health care centres will be invited to participate in the study. The patients will be recruited by health care professionals during their normal appointments.

**Table 1:** Statistics of health care regions

			Public Health Care		Occupational Health Care Enterprises	
Region	Population	18–64-year-olds (%)	Physicians (N)	Physiotherapists (N)	Physicians (N)	Physiotherapists (N)
ESSOTE	79,808	57.2	36	12	18	13
EKSOTE	131,764	58.6	58	28	10^a^	5^a^
Rovaniemi	62,420	64.9^b^	35	10	***	***

ESSOTE (Etelä-Savon sosiaali- ja terveystoimi, The South Savo social and health care authority), EKSOTE (Etelä-Karjalan sosiaali- ja terveyspiiri, South Karelia social and health care district)

^a^The figures include only those working in EKSOTE’s own occupational health service organization; there are four other occupational health service organizations in the South Karelia county. ^b^15–64-year-olds. (http://tilastokeskus.fi/index.html). ***No occupational health services involved in the study

### Eligibility criteria

All patients aged 18–65 who contact health care due to LBP, either with or without radicular pain, will be included in the study. Exclusion criteria are: 1) Age under 18 or over 65; 2) First patient-reported contact with health care due to LBP and episode lasting less than 2 weeks; 3) Suspicion of a serious cause of LBP or LBP requiring urgent care. Patients will receive written information on the study. Only patients who sign the consent form will be included in the study.

### Intervention

The intervention implements the classification-based biopsychosocial approach to LBP patients in primary care. Table 2 describes the elements of implementation. We will use the SBT for classification. The SBT consists of eight items (or themes): bothersomeness, referred leg pain, comorbid pain, disability (two questions), catastrophizing, fear, anxiety, and depressive symptoms. The response alternatives to Items 1–8 are “agree= 1 point” or “disagree = 0 point”. Item 9 has five options, of which the two highest responses will be counted as one point. Thus, the maximum total score range will be 0–9. In addition, the psychosocial subscale will be derived from Questions 5–9 (range 0–5). The following risk groups will be formed: 1) Low-risk (total score of 3 or less); 2) Medium-risk (total score of 4 or more and psychosocial subscale score of 3 or less); and 3) High-risk (total score and psychosocial subscale score of 4 or more) [39].

**Table 2:** Elements of implementation

Level of element	Implementation elements of a classification-based biopsychosocial approach
Organizations	Premeditated care pathways for LBP patients
	More resources targeted towards high-risk patients
	Support of electronic patient registry functions
Professionals	Education: Physicians 4 h, physiotherapists 4 days, nurses 2 h, short booster education sessions in units
	SBT used systematically
	Patient education booklet in use
	Premeditated phrases (and SBT) for nurses
	Referral to physiotherapy according to risk classification
Patients	SBT
	Patient education booklet
	Individual biopsychosocially-oriented care

*LBP* Low Back Pain, *SBT* STarT Back Tool

#### Organizational level

**Fig. 1:**
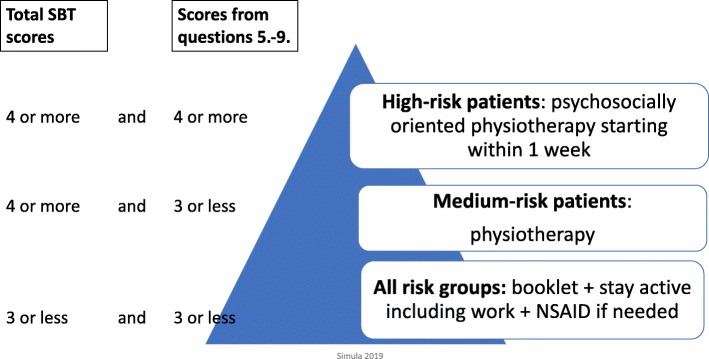
Aim of care pathway

We will evaluate all the possible pathways for the patients with LBP in each health care region. Direct access to a PT will be emphasized. For nurse appointments, premeditated phrases will be taught and integrated into the electronic patient record system. Local practices within each health care region will be modified to enable easy access to care according to risk classification. Local practitioners and managers such as the clinical director of the GPs, the nurse manager in primary care, and the manager of the PTs will be involved when modifying the pathways according to regional specific facilities and resources. All the health care professionals involved in the LBP patients’ care will be informed of the new care pathways. Education on the new strategies will be organized for professionals and written information will be given to each health care region. Figure 1 shows the aim of the care pathway and the detailed strategies for achieving it will be locally planned for each health care region. Table 3 describes the health care region-specific challenges and strengths during the implementation process. Co-operation between public health care and occupational health service organizations will be enhanced through joint education (not possible in Rovaniemi).

**Table 3:** Health care region-specific challenges and strengths during implementation process

Health care region	Challenges	Strengths
ESSOTE	A simultaneous extensive organizational change (fusion of primary and secondary health care organizations, including relocation of primary care facilities)	Research nurses and the principal investigator can remind/educate professionals of the new protocol from time to time. During the re-evaluation, additional education lessons will take place in units during • emergency duty nurses’ meeting 2x60min • student health care unit nurses’ meeting 1x90min • junior physicians’ meeting 1x75min • GPs’ meeting 1x60min • general medicine department nurses’ meeting 2x30min • PTs’ meeting 2x30min • occupational health physicians’ meeting • PTs’ meeting 1x60min The nurse in charge of the emergency room is active in improving the implementation of the new care strategy.
Rovaniemi	Low GP participation rate in education. Emergency department not part of the study. Simultaneous relocation of primary health care facilities. No occupational health service organization included the study.	Previously complicated wide criteria for direct access to PT enormously reduces the possibility to use it. A notable criterion for direct access to physiotherapy during implementation process might be helpful.
EKSOTE	Simultaneous change in electronic medical record system increases requirements to adopt new working practices among professionals.	Some biopsychosocial oriented education for PTs had been held before this study, which is helpful for implementation. Mentoring will be arranged for PTs during implementation and re-evaluation.

ESSOTE (Etelä-Savon sosiaali- ja terveystoimi, The South Savo social and health care authority), EKSOTE (Etelä-Karjalan sosiaali- ja terveyspiiri, South Karelia social and health care district)

#### Professional level

We will improve the implementation of the classification-based approach by educating professionals (nurses, PTs, physicians). The education seminar programmes are available in the supplementary information. Education will improve the implementation of the new care strategy and aims to enable professionals to equally transfer correct information from health care to all patients. The education will be provided by Professor of Physiatry, Jaro Karppinen, MD; Anna Sofia Simula, Specialist in General Medicine; Riikka Holopainen, Physiotherapist, MSc; Mikko Lausmaa, OMT Physiotherapist; and Maija Paukkunen, Occupational Physiotherapist. Jaro Karppinen is a specialist in Physical and Rehabilitation Medicine and has published over 200 peer-reviewed papers on musculoskeletal problems, including randomized controlled interventions among LBP patients. Anna Sofia Simula is an experienced clinician in the field of pain and has wide-ranging practical knowledge of health care services and organizations. All the trainers are experienced in using and teaching the biopsychosocial approach.

The PTs will receive 4 days (28 h) of education in biopsychosocially-oriented individualized physical therapy and classification-based care. The training will carefully cover the physiology of LBP from the biopsychosocial perspective, evidence-based care, and low value care. The main messages of the training will include a biopsychosocial explanation for pain, therapeutic alliance, validation and avoidance of invalidation, avoidance of unhelpful/harmful messages and unnecessary imaging in non-specific LBP. It will also contain an assessment of individual psychosocial factors (using SBT and the short version of Örebro Musculoskeletal Pain Screening Questionnaire) and individualized care plans in accordance with the risk classification, and demo patients’ cases. Listening and interaction will be highlighted. In the EKSOTE region, the PTs willing to treat high-risk patients will receive an extra half day of group training and personal mentoring for at least 3 months. Mentoring will include discussions in groups/pairs, accompanied by patient appointments of a colleague and goal-directed learning tasks related to their own work and clinical patients.

For physicians, we will provide a four-hour education session. Its four main themes will be: imaging issues, SBT, lifestyle factors, and work disability. We will discuss the relevance of lumbar MRI findings, such as the prevalence of findings among asymptomatic adults and the disadvantages of imaging. We will also teach key pain medication issues. The role of physiotherapy will be emphasized. The physicians will also be taught the main principles of biopsychosocially-oriented individualized care.

For the nurses we will conduct two two-hour sessions, which will include data on the natural course of LBP, the harm of nocebo messages that health care providers give patients, risk classification using the SBT and the current treatment principles and pathways. Premeditated phrases in electronic medical records will enhance patient history clarification and classification-based care guidance.

The professionals will systematically use the SBT with all their LBP patients and will make individual care plans for patients according to their risk profile. A patient education booklet, based on the biopsychosocial model, will be used to deliver evidence-based information on the aetiology of LBP and appropriate imaging to patients. It will also remind professionals of the biopsychosocial LBP model. The booklet has been validated and translated into Finnish (Simula et al. submitted).

#### Patient level

All the LBP patients will receive the patient-education booklet. They will be classified into low-, moderate- or high-risk groups during their first visit to primary health care, based on the SBT. The physicians and PTs will use the SBT as a classification method during the LBP patient’s visit. The physicians and PTs will plan the individual treatment process according to the risk classification.

Low-risk patients will receive advice on how to stay active including advice on pain medication, if appropriate. The patient education booklet will be based on the biopsychosocial model. Referral to a PT will only be scheduled when necessary.

Medium-risk patients will receive the same care as the low-risk group. However, all medium-risk patients will also be referred to physiotherapy. In addition to the clinical examination and patient advice, the PTs will evaluate their patients’ pain, fears and maladaptive behaviours. Physiotherapy will be individualized and at least one follow-up PT visit will be recommended. Each patient will have approximately two to 4 PT visits, but an individualized approach to care will be highlighted, so that exceptions can be made in each individual care plan. Co-existing symptoms will be evaluated and treated if needed. In cases of sleep problems, the patient will be referred to a sleep management group or individual guidance with a focus on non-pharmaceutical treatments, or to a psychiatric nurse in the health centre if needed. Other co-morbidities such as smoking, overweight and type 2 diabetes will be taken into consideration and the patient will be referred for further care if needed.

High-risk patients will receive a similar treatment protocol to medium-risk patients, but with an emphasis on psychosocial factors and with as short a delay a possible before starting physiotherapy (less than 1 week). Faster access to physiotherapy will be arranged using premeditated pathways for high-risk patients.

### Primary outcome measure

The primary outcome will be change in PROMIS PF-20 (Patient-Reported Outcomes Measurement Information System) (short form 20a) from baseline to 12-month follow-up. A translated and validated Finnish version of the PROMIS PF-20 will be used [46, 47].

### Secondary outcome measures

Pain and disability: change in PROMIS PF-20 from baseline to three-month follow-up; change in Oswestry Disability Index (ODI) from baseline to 3- and 12-month follow-up; change in SBT from baseline to 12-month follow-up.

Health-related quality of life: change in EQ-5D (EuroQol five dimensions) from baseline to 12-month follow-up.

Direct and indirect costs will be measured over the 12-month follow-up period, and will include:

- Direct costs: Physician visits, PT visits, nurse visits, other health care clinician visits (e.g. psychologist), imaging due to LBP (x-ray/MRI/CT (computer tomography)), pain medication, and surgical and other invasive interventions.
- Indirect costs: Days on sick leave (LBP-related and all sick leaves).

Details on prescription medicine reimbursements, as well as details on sick-leave payments are available from the nationwide registers maintained by the Social Insurance Institution of Finland (SII, Kela in Finnish). These should provide, to the nearest cent (¢), the costs paid by the health care sector in Finland for these two cost drivers. For the costs of visits to publicly provided health care we intend to use the information that the Finnish Institute for Health and Welfare (THL) gathers from Finnish hospitals, available from the Care Register for Health Care (CRHC). Secondary outcomes are described in Table 4.

**Table 4:** Secondary outcomes

**Pain and disability**	
Oswestry Disability Index, change from baseline to 3- and 12-month follow-up Roland Morris disability questionnaire, change from baseline to 12-month follow-up	
PROMIS (Patient-Reported Outcomes Measurement Information System) (short form 20a), change from baseline to 3-month follow-up	
Frequency of LBP during last 3 months, change from baseline to 3- and 12-month follow-ups	
LBP intensity (0–10 numerical rating scale (NRS)) during last week, change from baseline to 3- and 12-month follow-ups	
Leg pain intensity (NRS) during last week change from baseline to 3- and 12-month follow-ups	
SBT (STarT Back Tool) change from baseline to 12-month follow-up	
**Health-related quality of life**	
EQ-5D (EuroQol five dimensions) change from baseline to 12-month follow-up	
**Direct costs**	
Physician visits during last year	
Physiotherapist visits during last year	
Nurse visits during last year	
Other health care professional visits (e.g. psychologist) during last year	
Imaging due to LBP (x-ray/MRI/CT) during last year	
Pain medication during first year	
Back surgery rate	
**Indirect costs**	
Days on sick leave during last year (LBP-related and all sick leaves)	
Disability pensions during first year	

### Participant timeline

**Fig. 2:**
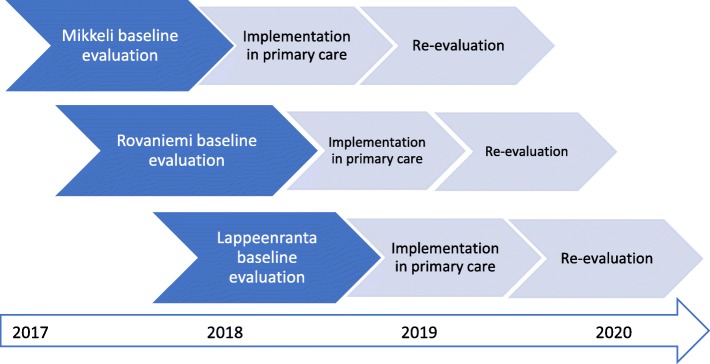
Flow chart of intervention

The baseline evaluation of the health care regions will contain organizational data, professional data, and patient-level data (described later). Approximately 1 year after baseline evaluation, we will implement a classification-based biopsychosocial model for the LBP patients in the study’s health care regions (Fig. 2). After implementation, the health care regions will be re-evaluated in a similar manner to that at baseline. In each region, data before the implementation will be compared to data after the implementation.

### Sample size

The primary outcome measure for this trial is the change in PROMIS from baseline to 12-month follow-up. Our sample size calculation is based on the following hypothesis test: superiority of a classification-based approach to LBP in primary care compared to best current care. For sample size calculation, we will use G*Power 3.1. (Difference between two dependent means). The Minimal Important Difference (MID) for PROMIS PF-20 change is about 2 points, Standard Deviation (SD) is 3.66 [48]. The effect size is 0.5, and type I error rate 0.05. A sample size of 34 patients per group (health care region) would enable the detection of a difference of 2 points in PROMIS with 80% power. With a 40% drop-out rate and 6 groups (3 before and 3 after), the final sample size is 340 patients.

### Recruitment

All GPs and PTs in the study health care centres will be invited to participate in the study. In all the centres, the clinicians (nurse, PT or physician) will receive 30 min of training on the study and eligibility criteria. After this, they will identify patients when consulted for LBP*.* The clinician will inform the identified patient of the study, give them patient information and ask for their consent. In most cases, the first contact and recruiting clinician is a nurse or PT, even if the patient also has a physician appointment, and this should reduce selection bias. Signed consent forms will be sent to the research nurse (out of clinic). The physicians and PTs will be informed and reminded monthly about the study via email. We will use the same recruiting strategy before and after the implementation of the classification-based approach.

### Data collection, management and analyses

We will collect organizational data, professional data and patient-level data (Table 5). Organizational administrative register data will be collected directly from each health care organization and will contain no identifiable patient information. The first professional data collection will be conducted after the professionals have undergone a 15–30-min study information session. The research nurse will send a web-based questionnaire to all the physicians and PTs in the study health care units who have consented to participate in the study (Table 6). Professional data will be collected from each health care unit and will contain no identifiable individual practitioner data.

**Table 5:** Evaluation of health care region

Domain	Measures
**Organizational**	Number of physician appointments according to ICD10 M40-M53
	Imaging examinations
	Secondary care consultations due to LBP
**Professional**	Professionals’ beliefs and attitudes to LBP
**Effectiveness of LBP care**	Patient data (outcomes described in Table 6)
**Cost-effectiveness**	Direct costs
	Indirect costs

**Table 6:** Components of professional data collection

Domain	Measures
**Descriptive data**	Gender
	Occupation (physician/physiotherapist/occupational nurse/other)
	Clinical work experience
	Health care unit for work
	Proportion of patients with LBP of all consultations
**Attitudes and beliefs**	Back pain beliefs questionnaire
	ABS-mp (Attitudes to Back Pain Scale, for musculoskeletal practitioners)
**Routines and satisfaction**	Use of patient education leaflet
	Use of risk stratification tool in practice (e.g. SBT)
	Satisfaction with treatment
	Level of confidence in own skills

Patient- level data will be collected through baseline and follow-up surveys at 3 months and 1 year via web-based questionnaires. The research nurse will email the patients the baseline questionnaire link after receiving their signed consent. We will aim to have the responses to the baseline questionnaires between one and 3 weeks after the consultation in the research health care unit. If an email address is missing, the research nurse will call the patient to acquire it or send them a paper version of the questionnaire. The research nurse will resend the link 1 week later if no response is received. After 2 weeks, and again after 3 weeks, the research nurse will remind the patients who have not answered and will send them a text message with a hyperlink to the questionnaire. The patients’ questionnaire includes descriptive data and the validated symptom-related questions listed in Table 7.

**Table 7:** Components of patient-level data collection

Domain	Measures	Time Point (months)
**Descriptive data**	Age and gender	0
	Occupation	0
	Weight and height	0
	Country of birth	0
	Pregnancy	3, 12
**Lifestyle**	Leisure time physical activity	0
	Smoking	0, 12
**Comorbidity**	Diabetes, rheumatoid arthritis, ankylosing spondylitis, osteoarthritis, depression, fibromyalgia, inflammatory bowel disease, muscle disease	0
**Back pain**	Previous back pain episode of at least two weeks’ duration	0
	Previous (lifetime) physician consultations related to back pain	0
	Frequency of LBP during last three months	0, 3, 12
	LBP intensity during last week, using 0–10 scale NRS (Numeral Rating Scale)	0, 3, 12
	Leg pain intensity during last week, using 0–10 NRS	0, 3, 12
**Work status**	Employment/unemployment/pension/student/unpaid work at home/other	0
	LBP-related sick leave during last three months	0, 3
	LBP-related sick leave during last nine months	12
	LBP-related part-time sick leave during last three months	0, 3
	LBP-related part-time sick leave during last nine months	12
	Work modifications due to LBP	0, 3, 12
**Use of health care resources**	Physician consultations during last three months	0,3
	Physician consultations during last year	12
	Physiotherapist consultations during last three months	0,3
	Physiotherapist consultations during last year	12
	Nurse consultations during last three months	0,3
	Nurse consultations during last year	12
	Other health care clinician consultation (e.g. psychologist, occupational therapist) during last three months	0,3
	Other health care clinician consultation (e.g. psychologist, occupational therapist) during last year	12
	Imaging due to LBP (x-ray/MRI /CT) during last year	0, 12
	Imaging due to back pain (x-ray/magnetic resonance imaging/computed tomography) during last three months	3
	Referral for imaging examinations (x-ray/MRI/CT) due to back pain	0, 3, 12
**Medication**	Over-the-counter pain medication during last week	0, 3,12
	Prescription pain medication (paracetamol/anti-inflammatory/mild opioid/strong opioid/others)	0, 3, 12
**Surgery**	Spine operation	12
**Patient satisfaction**	With information related to pain explanation	0, 3, 12
	With self-efficacy	0, 3, 12
	With health care provider’s skills	0, 3, 12
	With being heard and understood in terms of symptoms	0, 3, 12
**Pain and disability**	PROMIS PF-20 (Patient-Reported Outcomes Measurement Information System, 20-item physical functioning short form)	0, 3, 12
	STarT Back Tool	0, 12
	Örebro Musculoskeletal Pain Screening Questionnaire	0
	Oswestry Disability Index	0, 3, 12
	Roland Morris Disability Questionnaire	0, 12
**Beliefs**	FABQ (Fear avoidance Beliefs Questionnaire)	0, 12
	PSEQ (Pain Self-Efficacy Questionnaire)	0, 12
	BBQ (Back Beliefs Questionnaire)	0, 3
**Depressive symptoms**	DEPS (Depression Scale)	0
**Work ability**	Current work ability compared with lifetime best (0–10)	0, 3, 12
	Work ability in relation to demands of job	0, 12
	Estimated work impairment due to disease	0, 12
	Own prognosis of work ability two years from now	0, 12
**Health-related quality of life**	EQ 5D (EuroQol five dimensions)	0, 3, 12

*MRI* magnetic resonance imaging, *CT* computed tomography

### Statistical methods

Baseline characteristics will be analysed using descriptive statistics. The change in each outcome from baseline to the follow-up visits in the intervention regions and the differences between the intervention regions and control regions will be analysed using a range of statistical methods, including univariate and multivariate techniques. SPSS Statistics (version 25) will be used for statistical analyses. For the cost-effectiveness analysis we will use the health care resource questionnaires described earlier. Health care resource use will be calculated as the number of visits multiplied with the unit cost per item and expressed as mean costs by items of resource use and the mean direct total health care resource costs. All costs will be discounted to the price level of the most recent follow-up year.

### Monitoring

A data monitoring committee will be not needed because the study will be conducted by independent researchers and no sponsors with competing interests will be involved. The only potential negative outcome of participating in the study is the time lost while answering the web-based questionnaires. No compensation will be paid for answering the questionnaires, and failing to answer them will not be reported to the health care professionals responsible for treating the patients. A research nurse will collect the data weekly.

## Discussion

LBP is one the most prevalent and disabling health conditions. Its economic burden to society is enormous. For many LBP patients, the symptoms tend to be prolonged and become chronic partly due to lack of a biopsychosocially-oriented approach to treatment in primary health care. From the individual’s as well as society’s perspective, it is important to create evidence-based and cost-effective treatments for LBP patients, especially in early phases of LBP, in order to prevent pain becoming chronic and causing work disability. In this study, we extend the intervention to the entire care pathways, including the organization, professionals and the individual LBP patients themselves. A multidimensional intervention may enhance the implementation of the classification-based approach and the effectiveness of care. BCT is the only design able to assess the effectiveness of the entire care pathway in routine health care [44]. By including three different health care regions we will be able to evaluate the differences before and after the intervention in each one and between the health care regions. We will also able to detect the best practice or unsuccessful implementation by a combination of organizational, professional and patient-level data. For patient-level data, we will use the recommended outcome measurement instruments: ODI for physical functioning, NRS for pain intensity, PROMIS for pain and disability, and EQ. 5D for health-related quality of life [49]. The results of the trial will be published in peer-reviewed international journals, and disseminated through both conventional media and social media.

## Supplementary information

**Additional file 1.**

## Data Availability

Seminar programs are available in additional file. Patient data are not publicly available due to this being an ongoing study.

## Abbreviations

SBT: The STarT Back Tool
LBP: low back pain
BCT: Benchmarking Controlled Trial
PROMIS: Patient-Reported Outcomes Measurement Information System (short form 20a)
MRI: Magnetic resonance imaging
PT: Physiotherapist
GP: General practitioner
ESSOTE: (Etelä-Savon sosiaali- ja terveyspalvelut, The South Savo social and health care authority)
EKSOTE: (Etelä-Karjalan sosiaali- ja terveyspiiri, South Karelia social and health care district)
ODI: Oswestry Disability Index
EQ-5D: EuroQol five dimensions
CT: Computer tomography
SII, Kela in Finnish: The Social Insurance Institution of Finland
THL: The Finnish Institute for Health and Welfare
CRHC: The Care Register for Health Care
MID: Minimal Important Difference
SD: Standard Deviation
NRS: Numerical rating scale
FABQ: Fear avoidance Beliefs Questionnaire
PSEQ: Pain Self-Efficacy Questionnaire
BBQ: Back Beliefs Questionnaire
DEPS: Depression Scale
