# Supplementary material for: A classification-based approach to low back pain in primary care – protocol for a benchmarking controlled trial
Source: BMC Fam Pract. 2020 Apr 6;21:61. doi: 10.1186/s12875-020-01135-8 (PMC7137427; doi:10.1186/s12875-020-01135-8)
Supplement: Supplementary file 1 — Additional file 1. [file 12875_2020_1135_MOESM1_ESM.pdf]

Trust your back – seminar Mikkeli

Date: **Part 1: 21st to 22nd March 2018; Part 2: 19th to 20th April 2018.**

Place: **MKS (Mikkeli Central Hospital), video connection available**

Organizer: **Trust your back research team, ESSOTE (The South Savo Social And Health Care Authority), The Finnish Medical Society Duodecim, Local Department of Mikkeli**

The seminar is for all health care professionals involved in low back pain patients' care in primary care. We recommend that physiotherapists in particular attend the whole seminar. We recommend that medical doctors attend at least the lectures in green if they are unable to participate in the whole seminar.

21.3.2018

8-8:10 Welcome, Anna-Sofia Simula, MD (ASS)

8:10-9:00 Imaging in Low Back Pain, Professor Jaro Karppinen (JK)

9:00-9:45 Pain physiology, Placebo - Nocebo, ASS

9:45-10:00 Break

10:00-10:45 Psychosocial risk factors, MSc Physiotherapist Riikka Holopainen (RH)

10:45-11:15 Trust Your Back – Introduction to the research project, ASS

Lunch break

12:15-13:15 Therapeutic alliance and practitioners' beliefs, OMT physiotherapist Mikko Lausmaa (ML) and RH

13:15-14:15 Low back pain patient interviews using the cognitive behavioural approach, RH

14:15-14:30 Break

14:30-15-15 Fear-avoidance behaviour, ML

15:15-15:45 Discussion and closing of the the day

Sauna in Tönölä starting at 17.00.

22.3.2018

8-9:00 Lifestyle risk factors, JK

9:00-10:00 Examination of low back pain patient, ML

10:00-10:15 Break

10:15-11:15 Targeted care for low back pain patients, examples, ML

Lunch

12:15-14:15 Demo patient 1. appointment, RH

14:15-14:30 Break

14:30-15:15 Summary and clarification of demo patient appointment (and Interview training if time allows)

15:15-15:45 Discussion and closing of the day

19.4.2018

8-8:45 Evaluation of the risk using Keele STarT Back Tool and Örebro musculoskeletal pain screening questionnaire, research evidence, JK

8:45-9:15 How to explain pain to the patient. RH

9:15-9:30 Understanding low back pain – patient education booklet, ASS

9:30-9:45 Break

9:45-11:15 Demo patient 2. Appointment, ML

Lunch

12:15-13:15 Practising the interview, RH

13:15-13:45 Low back pain patient care, examples, ML

13:45-14:00 Break

14:00-15:15 Demo patient 1, reassessment, RH

15:15-15:45 Summary and clarification of the demo patient case

20.4.2018

8-8:45 Sick leaves, JK

8:45-9:30 Work-related interventions, Occupational Physiotherapist Maija Paukkunen (MP)

9:30-9:45 Break

9:45-10:30 How to implement the new care strategy in local practice. ASS

10:30-11:15 Low back pain with radiculopathy, ML

Lunch

12:15-14:15 Demo patient 3. Appointment, RH

14:15-14:30 Break

14:30-15:15 Summary and clarification of the demo patient case

15:15-15:45 Planning the local policy in groups with colleges from own health care unit, ASS, MP, RH, ML, JK

15:45-16:00 Discussion and closing of the seminar

Trust your back – seminar Rovaniemi

Date: **Part 1: 11th–12th December 2018; Part 2: 30th–31st January 2019**

Place: **Metsäruusuntie, Auditorium, video connection available (not during demo patients); Parallel lectures for nurses,** Rinteenkulma Health care unit, Meeting room.

Organizers: **Trust your back research team, Lapland Hospital District**

The seminar is for all health care professionals involved in low back pain patient care in primary care. We recommend that physiotherapists especially attend the whole seminar. We recommend that physicians attend at least some lectures on the programme if they are unable to participate in the whole seminar. The lectures especially for nurses are provided as parallel sessions.

**Tuesday 11th Dec**	**Physiotherapists and physicians**
8-8:20	Welcome, Trust Your Back – Introduction of the research project, MD Anna-Sofia Simula (ASS)
8:20-9:10	Imaging in Low Back Pain, Professor Jaro Karppinen (JK)
9:10-10:00	Lifestyle risk factors (JK)
Break	**Physiotherapists' programme continues after the break**
10:15-11:00	Introduction and professionals' beliefs. OMT physiotherapist Mikko Lausmaa (ML)
Lunch	
12:15-12:45	Pain physiology, Placebo – Nocebo (ASS)
12:45-13:30	Psychosocial risk factors, STarT Back Tool (SBT) and Örebro musculoskeletal pain screening questionnaire – short form (ÖMPSQ-short) (ML)
13:30-14:15	Clinical reasoning in LBP using biopsychosocial approach (ML)
Break	
14:30-15:15	Patients' beliefs; pain behavior; how to examine patients' performance (ML)
15:15-15:45	Discussion and closing of the day

**Wednesday 12th Dec**	**Physiotherapists**	**Nurses at 14-16**
8-8:45	Therapeutic alliance, Physiotherapist Riikka Holopainen, MSc (RH)	
8:45-9:15	Low back pain patient interviews using the cognitive behavioural approach (RH)	
Break		
9:30-11:15	Demo patient 1. Appointment (ML)	
Lunch		
12:15-13:15	Summary and clarification of demo patient appointment	
13:15-14:00	Targeted care for low back pain patients, examples (ML)	
Break		Pain and LBP 30min (ASS)
14:15-15:15	Training, key management principles (ML, JK)	Therapeutic alliance and validation 60min (RH)

15:15-15:45	Discussion of and closing the day	Patient education booklet 30min (ASS)

**Wednesday 30th Jan**	**Physiotherapists**	**Nurses at 14-16**
8-8:30	Understanding low back pain – patient education booklet (ASS)	
8:30-9:30	How to explain pain to the patient. Positive messages and validating interaction (RH)	
Break		
9:45-11:15	Demo patient 2. Appointment (RH)	
Lunch		
12:15-13:15	Summary and clarification of demo patient's appointment.	
13:15-13:45	Training, difficult interaction situations (ML, RH)	
Break		**Occupational physiotherapist Maija Paukkunen and ASS**
14:00-15:15	Demo patient 1, reassessment (ML)	SBT and training
15:15-15:45	Summary and clarification of the demo patient's appointment. Coming up questions.	Classification-based LBP care, local policy in Rovaniemi

**Thursday 31st Jan**	**Physiotherapists**
8-8:45	LBP with radiculopathy (ML)
8:45-9:30	New method and opportunities for LBP care in occupational health services (MP)
Break	
9:45-11:15	Demo patient 3. Appointment (ML)
Lunch	
12:15-13:15	Summary and clarification of the demo patient's appointment.
13:15-13:45	Training, plan for relapse (RH, ML)
Break	
	**Physiotherapists and physicians**
14:00-14:40	Risk evaluation using SBT and ÖMPSQ-short, research evidence, JK
14:40-15:10	Sick leaves (JK)
15:10-15:40	Classification-based LBP care, local policy in Rovaniemi (ASS)
15:40-16:00	Discussion and closing of the seminar

Trust your back – seminar Lappeenranta

The aim of the seminar is to implement evidence-based knowledge and practice for low back pain (LBP) care in routine health care. The theory will be applied using examples and training. Local policy will be defined to facilitate the classification-based care plan and individual management. The seminar is for general practitioners in primary care.

Date: Part 1: 18th to 19th September 2019; Part 2: 13th to 14th November 2019.

Place: IVH Campus, meeting room Suvorov. Laserkatu 6.

Organizer: Trust your back research team and EKSOTE (The South Karelia social and health care district)

Instructors: Professor Jaro Karppinen (JK), MD Anna-Sofia Simula (ASS), MSc Physiotherapist Riikka Holopainen (RH), OMT physiotherapist Mikko Lausmaa (ML) and Occupational physiotherapist Maija Paukkunen.

**18th Sep**	**Theme: interaction**
**8**	Trust Your Back – Introduction of the research project and aim of the seminar (ASS)
	Pain physiology, Placebo – Nocebo (ASS)
**9**	Beliefs (ML)
**break**	
**10**	Language (RH)
**11**	Interaction during an appointment (ML)
**lunch**	
**13**	Demo patient 1 appointment, ML
**14**	Summary and clarification of the demo patient's appointment.
**15**	Live training and explanation of independent task (ML)

**19th Sep**	**Theme: Clinical reasoning**
8-10.00 at health care unit, physicians' meeting, video connection to IVH Campus	
**8**	Trust Your Back – Introduction of the research project (ASS)
	Imaging in Low Back Pain (JK)
**9**	Lifestyle risk factors (JK)
**break**	

10 LBP patient examination (RH)

11 Clinical reasoning using biopsychosocial approach (ML)

lunch

13 Clinical reasoning using video of LPB patient’s appointment (ML)

14 How to explain or help the patient find the reasons their pain (ML)

15 STarT Back Tool (SBT). Sick leaves. (JK)

**13th Nov**	**Theme: Are expectations met?** (additions based on feedback is highlighted)
**8**	Run through of independent task. Recap of Part 1 of seminar using Kahoot®.<br>Why patients' and practitioners' beliefs are significant? (RH)
**9**	Managing acute LBP, time utilization (RH)
**break**	
**10**	Using the Patient education booklet at the appointment. Explaining pain and imaging issues, phrases in electronic medical record system, documentation, how to survive despite limited time resource. (ASS)
**11**	What we know about ergonomics (MP)
**lounas**	
**13**	Observing yourself and your colleague. Itsen ja kaverin havainnointia (MP)
**14**	Patient guidance. Clinical reasoning + instructions to help. Training. (RH)
**15**	Dramatized DEMO appointment

**14th Nov**	**Theme: patients' path**
**8**	Classification-based LBP care, local policy in EKSOTEN (ASS)<br>What does the new strategy mean in my unit? Workshop within own unit colleagues.
**9**	Research project in the future. (ASS)
**break**	
**10**	New method and opportunities for LBP care in occupational health services (MP)
**11**	Co-operation between public health care and occupational health services, workshop (MP)
**lunch**	
**13**	Individualized care plan. Training, PTA, SBT/ÖMPSQ-short repetition
**14**	Training (RH)
**15**	Professional learning plan. Smart. Resources. (RH)
